# An epigenome-wide association meta-analysis of prenatal maternal stress in neonates: A model approach for replication

**DOI:** 10.1080/15592294.2016.1145329

**Published:** 2016-02-18

**Authors:** Jolien Rijlaarsdam, Irene Pappa, Esther Walton, Marian J. Bakermans-Kranenburg, Viara R. Mileva-Seitz, Ralph C.A. Rippe, Sabine J. Roza, Vincent W.V. Jaddoe, Frank C. Verhulst, Janine F. Felix, Charlotte A.M. Cecil, Caroline L. Relton, Tom R. Gaunt, Wendy McArdle, Jonathan Mill, Edward D. Barker, Henning Tiemeier, Marinus H. van IJzendoorn

**Affiliations:** a Centre for Child and Family Studies, Leiden University, Leiden, the Netherlands; b Generation R Study Group, Erasmus MC-University Medical Center Rotterdam, Rotterdam, the Netherlands; c Department of Child and Adolescent Psychiatry/ Psychology, Erasmus MC-University Medical Center Rotterdam, Rotterdam, the Netherlands; d School of Pedagogical and Educational Sciences, Erasmus University Rotterdam, Rotterdam, the Netherlands; e Institute of Psychiatry, Psychology and Neuroscience, King's College London, London, UK; f Department of Psychiatry, Erasmus MC-University Medical Center Rotterdam, Rotterdam, the Netherlands; g Department of Epidemiology, Erasmus MC-University Medical Center Rotterdam, Rotterdam, the Netherlands; h Department of Pediatrics, Erasmus MC-University Medical Center Rotterdam, Rotterdam, the Netherlands; i Medical Research Council Integrative Epidemiology Unit, University of Bristol, Bristol, UK; j School of Social and Community Medicine, University of Bristol, Bristol, UK; k University of Exeter Medical School, University of Exeter, Exeter, UK

**Keywords:** Birth cohort, cord blood, DNA methylation, epigenome-wide association study (EWAS), prenatal maternal stress

## Abstract

Prenatal maternal stress exposure has been associated with neonatal differential DNA methylation. However, the available evidence in humans is largely based on candidate gene methylation studies, where only a few CpG sites were evaluated. The aim of this study was to examine the association between prenatal exposure to maternal stress and offspring genome-wide cord blood methylation using different methods. First, we conducted a meta-analysis and follow-up pathway analyses. Second, we used novel region discovery methods [i.e., differentially methylated regions (DMRs) analyses]. To this end, we used data from two independent population-based studies, the Generation R Study (n = 912) and the Avon Longitudinal Study of Parents and Children (ALSPAC, n = 828), to (i) measure genome-wide DNA methylation in cord blood and (ii) extract a prenatal maternal stress composite. The meta-analysis (n_total_ = 1,740) revealed no epigenome-wide (meta *P* <1.00e-07) associations of prenatal maternal stress exposure with neonatal differential DNA methylation. Follow-up analyses of the top hits derived from our epigenome-wide meta-analysis (meta *P* <1.00e-04) indicated an over-representation of the methyltransferase activity pathway. We identified no Bonferroni-corrected (*P* <1.00e-06) DMRs associated with prenatal maternal stress exposure. Combining data from two independent population-based samples in an epigenome-wide meta-analysis, the current study indicates that there are no large effects of prenatal maternal stress exposure on neonatal DNA methylation. Such replication efforts are essential in the search for robust associations, whether derived from candidate gene methylation or epigenome-wide studies.

## Introduction

Exposure to maternal stress *in utero* can negatively affect development in later life.^1-4^ For example, prenatal exposure to maternal depressive symptoms^5^ and contextual stress (e.g., economic disadvantage)^6^ have been associated with increased risk for offspring problem behavior, beyond variance attributable to postnatal exposures. It is increasingly recognized that epigenetic mechanisms, such as DNA methylation, might help explain the link between prenatal exposure to maternal stress and adverse developmental consequences.^7,8^ The extent to which prenatal maternal stress exposure relates to offspring genome-wide DNA methylation at birth remains unclear.

The vast majority of studies investigating the association between prenatal exposure to maternal stress and offspring methylation at birth have focused on candidate genes.^9-14^ For example, Cecil et al.^9^ demonstrated that neonates who were exposed to maternal stress (e.g., maternal psychopathology, criminal behaviors, substance use) in the prenatal period had higher methylation levels of the oxytocin receptor (*OXTR*) gene than non-exposed neonates. Higher neonatal *OXTR* methylation, in turn, showed temporal stability (from birth to 9 y of age) and was associated with callous-unemotional traits at age 13 y, independently of postnatal stress exposure and associated *OXTR* methylation.^9^ Similarly, prenatal exposure to maternal depressive symptoms has been associated with altered methylation of the serotonergic *SLC6A4* gene,^10^ the glucocorticoid receptor (*NR3C1*) gene,^11^ and imprinted genes^12-14^ in neonates or infants. Overall, the findings of these candidate gene studies support the “fetal programming hypothesis,”^15^ suggesting that exposure of the fetus to maternal stress *in utero* may influence DNA methylation in genes involved in fundamental developmental processes.

Since strong prior biological knowledge of the complex association between prenatal maternal stress exposure and offspring differential DNA methylation is lacking, it is critical to perform hypothesis-free, epigenome-wide association studies (EWASs) in addition to candidate gene studies.^16,17^ The few EWASs that have investigated the association between prenatal maternal stress exposure and offspring DNA methylation suffer from small sample sizes with limited generalizability and they have produced conflicting findings.^18^ Non et al.^19^ reported an association between prenatal exposure to maternal depression and offspring DNA methylation in 36 mother-offspring pairs. However, using a larger but still modest sample of 201 neonates born to mothers receiving psychiatric care, Schroeder et al.^20^ reported that maternal depression during pregnancy was unrelated to neonatal DNA methylation. Another small EWAS (n = 36) provided evidence that prenatal exposure to a natural disaster (i.e., the Quebec ice storm in 1998) was associated with offspring methylation in multiple genes predominantly related to immune function.^21^ However, DNA methylation was measured eight to 13 y after exposure and it cannot be excluded that the observed DNA methylation patterns were associated with unmeasured long-lasting environmental factors that were related to the original natural disaster but occurred *after* the prenatal period. As such, EWASs must be conducted in large samples of neonates and their mothers before more definite conclusions can be reached.

The aim of this study was to examine the association between prenatal exposure to maternal stress and offspring genome-wide cord blood methylation using different methods. First, we conducted a meta-analysis and follow-up pathway analyses. Second, we used novel region discovery methods [i.e., differentially methylated regions (DMRs) analyses] that are tailored to the Illumina Infinium HumanMethylation450 BeadChip array^22^ but are not designed for meta-analysis. To this end, we used data from two independent population-based studies, the Generation R Study (n = 912) and the Avon Longitudinal Study of Parents and Children (ALSPAC, n = 828), to (i) measure genome-wide DNA methylation at birth (via cord blood), when it is not confounded by the effects of stressful postnatal conditions, and (ii) extract a prenatal maternal stress composite. The fact that ALSPAC and Generation R are highly compatible enabled us to study 450K HumanMethylation neonatal methylation in similar populations and use a similar phenotype definition. Of note, although the 450K HumanMethylation array is considered a highly suitable platform for large-scale studies, it targets only <2% of the CpG sites present in the human genome.^17^ The current study is one of the largest in this emerging field of EWAS, and the built-in meta-analysis and follow-up analyses might serve as a model for future studies.

## Methods

### Study design and participants

This study used data from two population-based cohorts, the Generation R Study and the Avon Longitudinal Study of Parents and Children (ALSPAC). Generation R is an ongoing epidemiological study of children born from 9,778 pregnant women residing in Rotterdam, the Netherlands, with expected delivery dates between April 2002 and January 2006. The design and sample characteristics of the Generation R Study have been described in detail elsewhere.^23,24^ The study was conducted in accordance with the guidelines proposed in the World Medical Association Declaration of Helsinki and was approved by the Medical Ethical Committee of the Erasmus University Medical Center, Rotterdam. The current research used a subsample of 969 Caucasian Dutch neonates and their mothers drawn from the Generation R Focus Study, an ethnically homogeneous subsample nested within the Generation R cohort, who had epigenetic data at birth that successfully passed quality control.

ALSPAC is an ongoing epidemiological study of children born from 14,541 pregnant women residing in Avon, UK, with an expected delivery date between April 1991 and December 1992 (85% of eligible population).^25^ The sample is representative of the general population.^26^ More details of the available data are available in the study website through a fully searchable data dictionary (http://www.bris.ac.uk/alspac/researchers/data-access/data-dictionary/). Ethical approval was obtained from the ALSPAC Law and Ethics Committee as well as Local Research Committees. The current research used a subsample of 914 mother-offspring pairs drawn from the Accessible Resource for Integrated Epigenomics Studies (ARIES,^27^ www.ariesepigenomics.org.uk) nested within ALSPAC, who had cord blood methylation data available that successfully passed initial quality control, before carrying out additional quality steps (see below).

Except for the factor analysis on prenatal adversities, in which we used data from all participants, the present study only included participants who had complete data for prenatal maternal stress exposure and methylation data (Generation R: n = 912; ALSPAC: n = 828). In both studies parents signed written consent for participation.

### Measures

#### DNA methylation data

In Generation R and ALSPAC, 500 nanograms of DNA from cord-blood (birth, n_Generation R_ = 979; n_ALSPAC_ = 914) underwent bisulfite conversion using the EZ-96 DNA Methylation Kit (Zymo Research Corporation, Irvine, USA). The Illumina Infinium HumanMethylation450 BeadChip Kit (Illumina Inc., San Diego, USA) was used to measure DNA methylation at 485,577 CpG probes. In both datasets, initial quality control of data generated was conducted to determine the status of staining, extension, hybridization, target removal, bisulfite conversion, specificity, non-polymorphic and negative controls.

The Generation R sample included the 969 neonates who had DNA methylation data that passed quality control. All 49,564 probes identified as having (i) a single nucleotide polymorphism in the single base extension site with a frequency of > 1% in the GoNLv4 reference panel^28^ or (ii) a non-optimal binding (non-mapping or mapping multiple times to either the normal or the bisulfite-converted genome) were removed from the data set, leaving a total of 436,013 CpG probes for analysis.

In ALSPAC, DNA methylation data was only available in samples that passed initial control. Furthermore, samples (n = 25) or probes (n = 7,873) that failed additional quality control steps (>1 % probes/ samples with background detection *P*-value >= 0.05) were excluded from further analyses. In ALSPAC, participants with non-Caucasian or missing ethnicity (based on self-reports, n = 61) were removed prior to the analysis. This left a total of 828 samples and 477,704 probes after quality control.

Both samples were normalized using the *dasen* method described by Pidsley et al.^29^ and dye bias corrected.^30^ Normalized values are β-values, which represent the methylation level at a CpG probe for each neonate. Last, only probes (n = 429,074) that were present in both Generation R and ALSPAC datasets (i.e., probes that passed quality control in both data sets) were included in all analyses.

#### Prenatal maternal stress exposure

A prenatal maternal stress exposure (PMSE) score had been previously created in ALSPAC based on maternal reports, covering four stress domains: (i) life stress (e.g., death in family, illness, work problems), (ii) contextual stress (e.g., financial difficulties, housing problems), (iii) personal stress (e.g., psychopathology, substance abuse including alcohol and drugs), and (iv) interpersonal stress (e.g.,, family relationship difficulties, arguments with partner).^9^ ALSPAC and Generation R have highly compatible measures enabling us to create a similar PMSE score in Generation R, based on maternal reports. For each domain, items were summed and divided by the number of completed items. Inter-correlations between the risk domain scores were positive (all *P <* 0.001). Confirmatory factor analysis (CFA) in Mplus 7.11^31^ was used to assess the internal reliability of the stress domains and to extract one PMSE score in the whole Generation R cohort, showing good model fit (RMSEA; acceptable fit ≤ 0.08; CFI and TLI; acceptable fit ≥ 0.90).^32,33^ See Supplementary Material for full item descriptions (Table S1), inter-correlations between the stress domains (Table S2), and the confirmatory factor analysis model and fit indices (Fig. S1).

In both studies, the PMSE score was logarithmic (base 10) transformed to approximate a normal distribution. Additionally, the transformed PMSE score was translated into z scores and screened for outliers (values were Winsorized when z-score ≥ 3.29, affecting n = 8 measurements in Generation R and n = 5 in ALSPAC). These analyses were conducted in SPSS 21.0 statistical package.^34^

#### Covariates

We adjusted for technical covariates, including the sample's array number and position on the array. Following the methods developed by Houseman et al,^35^ we included estimated proportions of cells in whole blood [proportion of CD8+ T-cells, CD4+ T-cells, natural killer (NK) cells, B-cells, monocytes and granulocytes] to adjust for different cell type compositions. Because the cell type proportions add up to approximately 100%, granulocytes were excluded to avoid multicollinearity.

Furthermore, we included as covariates child sex, gestational age at birth, and maternal smoking during pregnancy. In Generation R, information on child sex was obtained from midwife and hospital registries at birth. Gestational age at birth was established by fetal ultrasound examination. Information on maternal tobacco smoking was obtained by postal questionnaires in early, mid, and late pregnancy. Maternal smoking was categorized on the basis of all three questionnaires into “never smoked during pregnancy,” “quit as soon as pregnancy was known,” and “continued smoking during pregnancy.” In ALSPAC, information on child sex and gestational age at birth was obtained from self-reports, health and administrative records. Information on maternal tobacco smoking was obtained by self-reported questionnaires in early, mid, and late pregnancy.

### Statistical analysis

#### Epigenome-wide association study (EWAS) and meta-analysis

First, single probe analyses investigating the association between the PMSE score and neonatal DNA methylation were performed for the CpG probes (n = 429,074) that passed quality control in both the Generation R and ALSPAC cohorts. We used a linear mixed effects model, adjusting for fixed (i.e., gestational age, sex, maternal smoking during pregnancy, cell types estimation) and random (i.e., the sample's array number and position on the array) covariates.

Second, to maximize power to detect an association of the PMSE score with DNA methylation in neonates, we combined the data from the two independent samples in an EWAS meta-analysis. In this study, we performed both fixed- and random-effects, inverse-variance meta-analysis using the methods implemented in METAL (Meta-Analysis Helper),^36^ and METASOFT,^37^ respectively. We also investigated evidence of heterogeneity in the Generation R and ALSPAC data using the *I*^*2*^*-* statistic,^38^ considering only results with no strong evidence of heterogeneity (Heterogeneity *P* > 0.05) for further analysis. Of note, the power to detect heterogeneity is limited with small numbers of studies. Bonferroni (*P* = 1.00e-07) and FDR corrections were applied as epigenome-wide thresholds.

We conducted a sensitivity EWAS of extreme groups in Generation R to test the possibility that only extreme prenatal maternal stress would lead to differential methylation. Specifically, we dichotomized PMSE according to the 10% highest (n = 91) and the 10% lowest (n = 82) scores and used this binary variable in a discovery EWAS.

#### Correlation test

A correlation test was used to investigate the linear relationship of the effect sizes of CpG probes between the Generation R and ALSPAC samples. To this end, all CpG probes showing an association at a nominal threshold level (meta *P <* 0.05) in our EWAS meta-analysis were selected and the Pearson correlation of the effect sizes between the Generation R and ALSPAC samples was estimated. We also considered the sign of the regression coefficients.

#### Epigenome-wide pathway analyses

To investigate whether specific pathways are over-represented in our EWAS meta-analysis results, epigenome-wide pathway analyses were performed. For this purpose, all top hits derived from the EWAS meta-analysis (meta *P* < 1.00e-04) were selected and annotated to genes, using the IlluminaHumanMethylation450kCHR36 file. We used 3 different tools to sort these genes into pathways: (i) the PANTHER 9.0 pathway-classification tool,^39^ using the Gene Ontology (GO)-Slim Molecular Function annotation dataset,^40^ (ii) DAVID (Database for Annotation, Visualization, and Integrated Discovery),^41^ and (iii) GeneMANIA,^42^ based on GO terms. These methods adjust for differential gene size but not the unusual distribution of 450K HumanMethylation probes with respect to the gene. It is recommended to use multiple tools when conducting pathway analysis, to overcome methodological challenges specific to each tool.^43^

#### Differentially methylated regions (DMRs) analysis

Differentially Methylated Regions (DMRs) are defined as at least two spatially contiguous probes within 1 kb distance of each other and with a differential statistic consistently less than the 5th (for negative associations) or more than the 95th percentile (for positive associations).^44^ To identify DMRs associated with PMSE in neonates, we first used the *clusterMake*r function of the *bumphunter* package^45^ to assign CpG probes into clusters, separated with a maximum distance of 1kb within the Generation R sample. For each cluster, we used the GlobalTest statistical package^46^ to test the association between prenatal stress and differential methylation of each of these clusters, using a linear regression model, while adjusting for covariates. Finally, the top clusters identified in the Generation R sample were tested for association with PMSE in the ALSPAC sample, using the same linear regression model in the GlobalTest statistical package.

## Results

### Sample characteristics

The sample characteristics for all mothers and children participating in this study are presented in Table 1. The distribution of mean cord blood methylation values (β-values) in Generation R (n = 912) and ALSPAC (n = 828) is presented in Table 2.

**Table 1.** Sample characteristics.

		
	Generation R (n = 912)	ALSPAC (n = 828)
*Child characteristics*		
Sex, % girls	48.4	48.9
Gestational age, weeks	40.2 (1.43)	39.6 (1.50)
*Maternal characteristics*		
Age, years	31.7 (4.13)	29.56 (4.39)
PMSE score, log transformed	0.15 (0.11)	0.25 (0.14)
Smoking during pregnancy, %		
never	77.5	87.1
until pregnancy was confirmed	7.7	3.4
continued	14.8	9.5

* Values represent mean (standard deviation), unless otherwise specified.

**Table 2.** Summary of the number of CpGs and their proportional distribution according to mean methylation (β-value) density in the Generation R and ALSPAC samples.

	Mean β-value in cord blood			
*Generation R*	<0.3	≥ 0.3 to 0.7	≥ 0.7	Total
CpGs (n)	181,765	51,296	202,952	436,013
CpGs as proportion of total array (%)	42	11	47	100
*ALSPAC*				
CpGs (n)	187,967	69,082	220,655	477,704
CpGs as proportion of total array (%)	39	15	46	100

### Epigenome-wide association analysis meta-analysis

**Figure 1.**
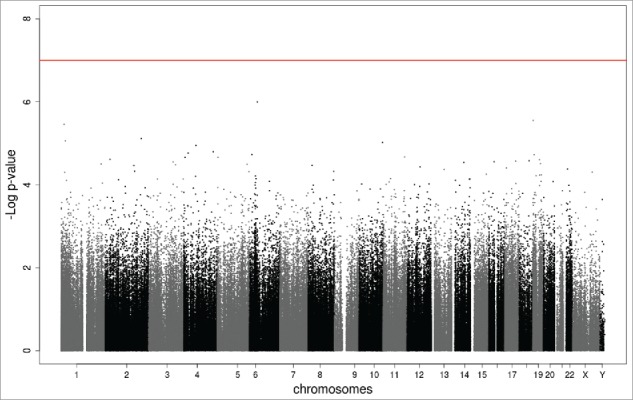
Manhattan plot of the EWAS meta-analysis of the PMSE score in cord blood (n_total_ = 1,740). The x-axis represents the autosomal (1–22) and sex (X,Y) chromosomes and the y-axis shows the –log_10_(*P*). The red line indicates the Bonferroni-corrected epigenome-wide threshold (*P* = 1.00e-07).

**Figure 2.**
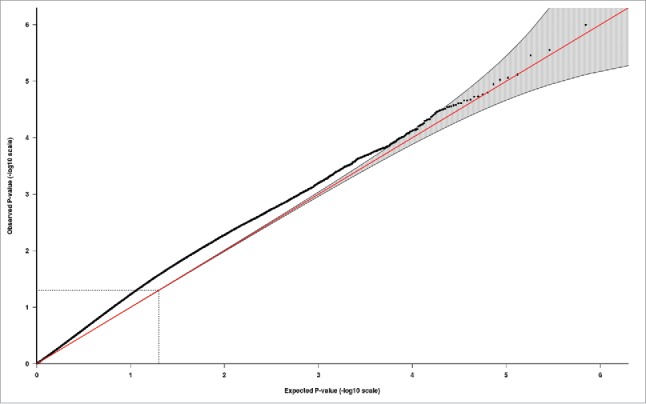
Quantile-quantile (QQ) plot illustrating probability values from the EWAS meta-analysis of the PMSE score in cord blood (n_total_ = 1,740). The red line indicates the distribution under the null hypothesis and the shaded area indicates the 95% confidence band.

Both the individual study epigenome-wide analyses and the meta-analysis (n_total_ = 1,740) revealed no epigenome-wide (Bonferroni- or FDR-corrected *P*-value) association of the PMSE score with neonatal DNA methylation. The top 10 CpG probes derived from the fixed-effects EWAS meta-analysis are presented in Table 3. More detailed tables with all top CpG probes (*P* < 1.00e-04) derived from the EWAS in the Generation R and ALSPAC samples, as well as the EWAS meta-analysis in the total sample, can be found in the Supplementary Material (Tables S3-S5, respectively). The Manhattan and quantile-quantile plot of the EWAS meta-analysis are presented in Figs. 1, 2, respectively. The random-effects EWAS meta-analysis revealed similar results as the fixed-effects EWAS meta-analysis. All top CpG probes identified by the random-effects EWAS meta-analysis are presented in the Supplementary Material (Table S6). Finally, the n_CpGs_ = 39,308 probes with weak evidence of differential methylation in the EWAS meta-analysis (meta *P <* 0.05) showed a strong correlation across the two samples [r(39,308) = 0.75, *P <* 0.01]. Of these 39,308 CpG correlations, 38,000 (97%) went in the same direction across Generation R and ALSPAC.

**Table 3.** Top 10 CpG probes (meta *P* < 1.00e-04) derived from the EWAS meta-analysis of prenatal maternal stress exposure in neonates, sorted by ascending meta *P*-value (n_*total*_ = 1,740).

			Generation R (n = 912)		ALSPAC (n = 828)		Meta-analysis (n_total_ = 1,740)				
Probe name	Chromosome	Position	Effect (SE)	*P*	Effect (SE)	*P*	Effect (SE)	Direction^*^	Meta *P*	Het *P*	Nearest gene (s)
cg13529437	6	43607635	−0.04 (0.009)	3.31e-06	−0.03 (0.016)	1.15e-01	−0.04 (0.007)	—	1.00e-06	.47	*MAD2L1BP*
cg04129946	2	201753996	0.02 (0.006)	2.63e-04	0.02 (0.007)	9.71e-03	0.02 (0.004)	++	7.59e-06	.73	*PPIL3;NIF3L1*
cg20959676	1	23696021	0.01 (0.003)	6.54e-03	0.02 (0.005)	1.01e-04	0.01 (0.003)	++	8.67e-06	.09	*C1orf213;ZNF436*
cg17631424	4	69312514	−0.06 (0.016)	2.64e-04	−0.05 (0.022)	1.53e-02	−0.06 (0.013)	—	1.13e-05	.81	*TMPRSS11E*
cg19459675	4	166249239	0.02 (0.005)	7.99e-04	0.02 (0.006)	6.72e-03	0.02 (0.004)	++	1.59e-05	.76	*SC4MOL*
cg12947485	4	25310668	−0.04 (0.010)	6.78e-04	−0.04 (0.015)	5.84e-03	−0.04 (0.009)	—	1.71e-05	.75	*NA*
cg20011562	6	12749352	0.01 (0.003)	2.78e-02	0.01 (0.004)	9.98e-05	0.01 (0.002)	++	1.85e-05	.18	*PHACTR1*
cg02644494	19	6412686	0.02 (0.008)	2.30e-03	0.03 (0.009)	2.77e-03	0.03 (0.006)	++	1.88e-05	.82	*PVRL1*
cg01686933	11	119596104	−0.02 (0.008)	6.49e-03	−0.03 (0.008)	1.11e-03	−0.02 (0.006)	—	2.12e-05	.81	*NA*
cg00409356	5	1879525	0.05 (0.012)	5.86e-05	0.02 (0.013)	5.75e-02	0.04 (0.009)	++	2.17e-05	.17	*IRX4*

* input order: the Avon Longitudinal Study of Parents and Children (ALSPAC) Study, the Generation R Study.

The prenatal maternal stress exposure score was not standardized and findings cannot be directly compared across ALSPAC and the Generation R Study.

NA: not available.

The sensitivity EWAS of extreme groups (i.e., 10% highest vs. 10% lowest PMSE scores) in Generation R similarly revealed no epigenome-wide association of PMSE with neonatal DNA methylation (data available upon request).

### Epigenome-wide pathway analysis

Three different pathway analyses tools (i.e., PANTHER, DAVID, and GeneMANIA) were used to test for over-representation of specific pathways within the top CpG probes (meta *P* < 1.00e-04) identified by our EWAS meta-analysis. The methyltransferase activity pathway was enriched in both PANTHER (Bonferroni-corrected *P* = 0.002) and GeneMANIA (false discovery rate, FDR *P* = 4.76e-04), and marginally enriched in DAVID (FDR *P* = 0.06) after correction for the number of pathways tested. Note that the same genes can participate in different pathways, making them not completely independent. The output of the pathway analysis tools is presented in Table 4.

**Table 4.** Pathway analysis of top CpG probes (meta *P <* 1.00e-04) derived from the EWAS meta-analysis of prenatal maternal stress exposure in neonates (n_total_ = 1,740).

**Pathway tool**	**Output**			
*PANTHER GO-slim Molecular Function*	*Expected n*_*genes*_	*Observed n*_*genes*_	*Fold enrichment*	*Pathway enrichment P*
Methyltransferase activity	0.26	4	>5	0.002^$^
*DAVID*	*Count*	*Coverage*	*Enrichment score*	*Pathway enrichment P*
Methyltransferase activity	5	1.1%	2.46	0.06^*^
*GeneMANIA*			*Coverage*	*Pathway enrichment P*
Methyltransferase activity			7/87	4.76e-04^*^

$ PANTHER reports the Bonferroni method

* DAVID and GeneMANIA report the Benjamini-Hochberg (false-discovery rate, FDR) method.

### Differentially methylated regions (DMRs) analysis

In Generation R, n_total_ = 151,704 clusters were identified, of which n_>2_ = 49,091 clusters contained more than 2 probes. These 49,091 clusters were tested for an association with the PMSE score. This analysis identified no Bonferroni-corrected (*P* = 0.05/49,091 = 1.00e-06) DMRS associated with prenatal maternal stress exposure in the Generation R sample. However, three DMRs, located in 20q13.33, 7q33, and 17q25.1, showed marginal associations with prenatal maternal stress. These three top DMRs were taken forward for replication in the ALSPAC sample. None of these regions were associated with PMSE in the ALSPAC sample. The results of the DMR analysis are presented in Table 5. A detailed annotation table of the DMRs can be found in the Supplementary Material (Table S7).

**Table 5.** Top Differential Methylated Regions (DMRs) associated with prenatal maternal stress exposure in the Generation R (n =912) and ALSPAC (n = 828) samples.

Cluster Number	n CpGs	Chromosome	Start Position	End Position	*P*_Generation R_	*P*_ALSPAC_	Nearest gene(s)
90946	3	20	62948037	62948235	3.75e-06	0.72	*MYT1;LINC00266-1;CICP4*
135488	11	7	133811808	133812369	1.27e-05	0.52	*LRGUK*
66361	15	17	73974861	73976089	3.55e-04	0.63	*ACOX1; C17orf106*

## Discussion

This study followed a hypothesis-free epigenome-wide (EWAS) approach to identify novel differentially methylated sites in the cord blood of neonates exposed to prenatal maternal stress. Both the study-specific epigenome-wide analyses in two independent population-based cohort studies, Generation R (n = 912) and ALSPAC (n = 828), and the EWAS meta-analysis in the total sample (n_total_ = 1,740), failed to provide evidence of Bonferroni-corrected DNA methylation differences in the cord blood of children exposed to prenatal maternal stress. The correlation test of the CpG probes (n_CpGs_ = 39,308) showing an association at a nominal threshold level in our EWAS meta-analysis (meta *P <* 0.05), indicated convergence between the Generation R and ALSPAC samples [r(39,308) = 0.75, *P <* 0.01].

Even though the current EWAS has significantly more statistical power to detect effects than the previous EWAS by Schroeder et al,^20^ who reported no differential DNA methylation in 201 neonates born to mothers with a lifetime history of mood disorder, it still finds only weak evidence. Cao-Lei et al.^21^ reported that prenatal exposure to a natural disaster was associated with offspring differential methylation (n = 36) in several genes during childhood. Furthermore, Non et al.^19^ showed that DNA methylation differed between neonates exposed to non-medicated maternal depression or anxiety (n = 13) vs. unexposed neonates (n = 23). It is noteworthy that these studies differ in the operationalization (i.e., psychopathology vs. natural disaster) and the timing (i.e., life-time vs. acute) of stress exposure. According to a recent meta-analysis, the effect of prenatal maternal stress exposure on infant birth weight and gestational age may vary according to how stress is operationalized.^47^ In the current study, we used a prenatal maternal stress exposure construct that incorporated a variety of stress domains. It is possible that the effects of prenatal maternal stress exposure are more subtle than those of acute extremely stressful events (e.g., a natural disaster) and that larger sample sizes are needed to identify these small, but potentially biologically important DNA methylation differences in cord blood. Another possibility is that there is no genome-wide DNA methylation effect in neonates in the general population. Of note, our sensitivity analysis of extreme groups (10% highest vs. 10% lowest prenatal maternal stress) similarly revealed no Bonferroni-corrected hits, which further supports the results of our meta-analysis but was very underpowered to confirm if there is a threshold effect of prenatal stress exposure on neonatal DNA methylation.

Of the CpG probes that did not surpass epigenome-wide thresholds in our study, the top hit (cg13529437, meta *P* = 1.00e-06) was located in chromosome 6p21.1, in the *MAD2L1BP* gene. The product of this gene participates in the mitotic checkpoint complex, acting as a regulator of the cell cycle.^48^ In cell lines, the *MAD2L1BP* gene was found to be induced by stressful events, such as ionizing radiation.^49^ However, in light of the observed sub threshold effects, we would like to point out that the exact contribution of this gene in conditions of maternal stress exposure is not straightforward and warrants further investigation.

Exploratory follow-up analysis of the top CpG probes (meta *P* < 1.00e-04) identified by our EWAS meta-analysis indicated an over-representation of the methyltransferase activity pathway. Methyltransferases are a large group of enzymes with the ability to methylate their substrates.^50^ Among our top EWAS meta-analytic results, both DNA and protein-methyltransferases were found. These results support previous research indicating that the methyltransferases regulating gene expression through DNA or histone methylation may also be under epigenetic control.^51,52^ This fine-tuned regulation of the epigenetic machinery offers an attractive system for feedback regulation and/or escalation of the response to initial environmental stimuli. For example, it has been suggested that epigenetic gene regulation associated with the altered expression of DNA methyltransferases is responsive to prenatal stress exposure^53,54^ and involved in the pathophysiology of schizophrenia^55,56^ and mood disorders.^57^ Future molecular and animal studies are needed to test this hypothesized mechanism.

The differentially methylated regions (DMRs) analysis in the Generation R sample identified three clusters in 20q13.33, 7q33, and 17q25.1 showing marginal associations with prenatal maternal stress. However, we were unable to replicate these clusters in the ALSPAC sample. These results highlight a major challenge of epigenetic epidemiology, which is to disentangle which associations are sample-specific, stochastic, or replicable, and eventually generalizable.^17^

We argue that the stress domains used in the current study, more so than acute disaster-type stresses studied by Cao-Lei et al.,^21^ are potentially generalizable to the broader population. Natural disasters are less common than financial and relationship difficulties, at least in Western industrialized countries. These financial and relationship difficulties are potentially more amenable to interventions than natural disasters. Hence, understanding the molecular consequences could inform prioritization of interventions in public health strategy. Using a more normative range of prenatal stresses, the current study promises to have widespread implications. Because the magnitude of these associations in terms of cell biology is unclear, the nominally significant results could include biologically important associations even if the change in methylation is quite subtle. Additionally, it is possible that the different stress domains exert differential effects on neonatal DNA methylation, and should be tested independently. However, in our samples the separate stressors showed skewed distributions that might lead to false positive findings. The aggregate score of these stressors better approximated a normal distribution, and therefore was used in our statistical analyses to capture the stressful environment to which both the mother and the fetus had been exposed.

Another potential factor to be taken into account in future EWASs is the timing of the prenatal maternal stress exposure. In support of the fetal programming hypothesis, Tobi et al.^58^ showed in a recent EWAS that prenatal famine exposure in early gestation, but not in mid- or late gestation, is associated with DNA methylation. Findings of a recent EWAS by Richmond et al.^59^ suggest that, in contrast to the view that early pregnancy represents a critical time-window for influencing development, sustained exposure of the fetus to maternal smoking *in utero* is required to induce neonatal DNA methylation modifications. The prenatal maternal stress measures used in the current study spanned 12 to 30 weeks of pregnancy and most indicators were assessed only once during pregnancy. There is a need for longitudinal EWASs investigating the relative roles of the early, mid, and late intrauterine environment in stress-induced DNA methylation changes, in order to identify the targets and timing of intervention.

The present results should be interpreted in the context of three main limitations. First, our analyses are limited to cord blood samples with heterogeneous cell types. Although it has become common practice to adjust for the proportion of cell types in the blood in EWASs,^35^ this approach has been validated for adults and not for cord blood samples. Alternative methods are needed to assess cell mixture distribution in cord blood. Second, although it has been shown that blood samples are adequate proxies of DNA methylation in other tissues (e.g., the brain^60^), there is also accumulative evidence of tissue-specific DNA methylation.^61^ Future research could investigate the effects of prenatal maternal stress exposure in specific tissues and cell types. Finally, an inherent characteristic of EWAS is that methylation at the majority of the CpG probes measured by the Illumina 450K array are either fully methylated or unmethylated, showing low variation between individuals.^62^ These CpG probes may be irrelevant for gene-expression regulation, and it is as yet unclear how this (lack of) variation should be taken into account. The Bonferroni correction for multiple testing as applied in this study could be in fact too strict, and alternative methods [e.g., selection of intermediate (mean β ≥ 0.3–0.7) or variable (SD ≥ 0.05) methylated CpG probes,^62^ use of region discovery methods,^44^ or estimation of equivalent number of independent variables^63^], are currently under development and can increase the power to detect true associations.

The present population-based study is the first, to our knowledge, to examine the association between prenatal maternal stress exposure and offspring genome-wide cord blood methylation using meta-analysis and novel region discovery methods. Meta-analysis approaches are widely used in genome-wide association studies and have identified many genetic variants, which could not be revealed in the individual studies.^64^ This study may showcase that a straight-forward approach in EWAS can be unsuccessful in terms of finding significant associations for specific probes, and that more sophisticated ways should be employed in epigenetic studies, whenever possible. The technical/ methodological properties of methylation arrays (e.g., limited coverage of the genome, cross-hybridization^65,66^), combined with the fact that inherently small effect sizes have been observed in small samples without replication in independent samples or model systems (e.g., cell lines and animal models), raise concerns regarding the replicability of the findings presented in the literature.^17^

Combining data from two independent population-based samples of mothers and neonates in an EWAS meta-analysis, this study identified no single CpG probe with strong associations with prenatal maternal stress exposure. The search for differentially methylated regions across the genome was followed up with several complementary approaches, including a correlation test, DMRs, and pathway analyses. The extent to which the severity, duration, and timing of prenatal stress exposures associate with neonatal DNA methylation should be further investigated in large longitudinal studies with more extreme variations in relevant phenotypes.

## Supplementary Material

KEPI_A_1145329_s02.zip
