# Supplementary material for: An epigenome-wide association meta-analysis of prenatal maternal stress in neonates: A model approach for replication
Source: Epigenetics. 2016 Feb 18;11(2):140–9. doi: 10.1080/15592294.2016.1145329 (PMC4846102; doi:10.1080/15592294.2016.1145329)
Supplement: KEPI_A_1145329_s02.zip [file kepi-11-02-1145329-s001.zip › KEPI_A_1145329_s02.pdf]

Table S1. Prenatal maternal stress exposure items in the Generation R sample

	Valid Range	*M (SD)*	*Scale*	*Time Points*	*Measurement Unit*	
**Life Stress**	0.00-5.37	1.47 (1.20)	Life events scale	20-25w gestation	Dichotomous	Your partner or child died
n = 886			Life events scale	20-25w gestation		A friend or relative died
			Life events scale	20-25w gestation		Child, partner or relative was seriously ill
			Quality of life scale	30w gestation		You were admitted to hospital (>24 hours)
			Quality of life scale	20-25w gestation		You were ill (moderate or poor health)
			Life events scale	20-25w gestation		You lost your job
			Life events scale	20-25w gestation		You had problems at work or school
			Life events scale	20-25w gestation		You moved house
			Quality of life scale	20-25w gestation		You experienced vaginal bleeding
			Quality of life scale	30w gestation		You had a test to see if your baby was abnormal
			Life events scale	20-25w gestation		Your house or car was burgled
			Quality of life scale	12-20w gestation		This pregnancy was unplanned
			Pregnancy outcome questionnaire	12-20w gestation		You were often worried about the health of the baby
			Quality of life scale	30w gestation		You were unhappy about the obstetric care
			Pregnancy outcome questionnaire	12-20w gestation		You were often worried about the pregnancy
**Contextual Stress**	0.00-3.61	0.58 (0.93)	Quality of life scale	30w gestation	Dichotomous	Housing basic living (indequate heating, no washing machine, or no refrigerator)
n = 839			Quality of life scale	30w gestation		Housing defects (cold or draught, windows are damp inside, or walls or furniture are damp)
			Long lasting difficulties questionnaire	20-25w gestation		Housing adequacy (e.g., too small, lack of privacy)
			Long lasting difficulties questionnaire	20-25w gestation		You had a major financial problem
			Quality of life scale	30w gestation		Financial difficulties
			Life events scale	20-25w gestation		Your income was reduced
**Personal Stress**	0.00-1.41	0.16 (0.38)	Moral values questionnaire	20-25w gestation	Dichotomous	You were in trouble with the law
n = 902			Family background question	Pregnancy composite		Early parenthood (age mother < 19 years)
			Family background question	Pregnancy composite		Maternal education
			Brief symptom inventory	20-25w gestation		Psychopathology of mother
			Lifestyle questionnaire	20-25w gestation		Substance abuse
			Moral values questionnaire	20-25w gestation		Violence offence
			Moral values questionnaire	20-25w gestation		Public order offence
**Interpersonal Stress**	0.00-6.36	1.04 (1.44)	Long lasting difficulties questionnaire	20-25w gestation	Dichotomous	You argued with your partner
n = 884			Long lasting difficulties questionnaire	20-25w gestation		You had arguments with your family or friends
			Family background question	Pregnancy composite		Partner status (single)
			Family assessment device	20-25w gestation		Family affection problems
			Social circumstances questionnaire	20-25w gestation		Family size (>3)
			Life events scale	20-25w gestation		You were divorced
			Family assessment device	20-25w gestation		Family major problems: difficult making plans
			Family assessment device	20-25w gestation		Family major problems: do not accept each other
			Family assessment device	20-25w gestation		Family major problems: cannot talk about sadness
			Family assessment device	20-25w gestation		Family major problems: avoid talking about worries and problems
			Family assessment device	20-25w gestation		Family major problems: feel unaccepted
			Family assessment device	20-25w gestation		Family major problems: unpleasant and painful feelings
			Family assessment device	20-25w gestation		Family major problems: cannot solve problems
			Family assessment device	20-25w gestation		Family major problems: decision-making is a problem
			Family assessment device	20-25w gestation		Family major problems: do not trust each other
			Family assessment device	20-25w gestation		Family major problems: conflict, argued
			Family assessment device	20-25w gestation		Family support problems
			Long lasting difficulties questionnaire	20-25w gestation		Difficulties in contact with others

Confirmatory factor analysis model fit indices: (i) *life stress* : RMSEA=0.039; CFI=0.834; TLI=0.806; (ii) *contextual stress:* RMSEA=0.056; CFI=0.982; TLI=0.971; (iii) *personal stress* : RMSEA=0.026; CFI=0.941; TLI=0.911; (iv) *interpersonal stress* : RMSEA=0.051; CFI=0.922; TLI=0.912.

Table S2. Intercorrelations between the prenatal stress domains in the Generation R sample

	Life Stress	Contextual Stress	Personal Stress	Interpersonal Stress
Life Stress	-			
Contextual Stress	.22***	-		
Personal Stress	.16***	.26***	-	
Interpersonal Stress	.22***	.31***	.24***	-

*** $p < 0.001$

Figure S1. Confirmatory factor analysis measurement model of prenatal maternal stres exposure in the Generation R sample

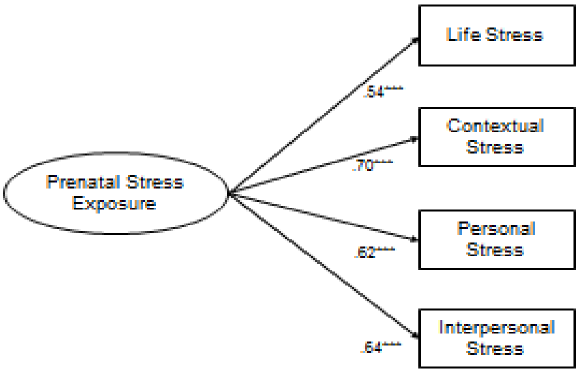

Note. Values represent standardized factor loadings for the confirmatory factor analysis of prenatal maternal stress exposure.
*** $p < 0.001$
CFA model fit indices $\chi^2(2) = 5.07$, $p = 0.079$;
RMSEA = 0.014; CF I= 0.999; TLI = 0.997

Table S3. Top CpG probes (p<1.00e-04) derived from the EWAS of prenatal maternal stress exposure in neonates, sorted by ascending p, in the Generation R sample (N = 912)

**Probe name**	***Effect***	***SE***	***p***	**Chromosome**	**Position**	**Nearest gene**
cg25937149	-.01	.001	2.10e-07	5	175788949	*KIAA1191*
cg19510604	-.03	.006	1.83e-06	15	74901076	*CLK3*
cg13529437	-.03	.008	3.31e-06	6	43607635	*MAD2L1BP*
cg27331471	.07	.015	5.34e-06	19	45809725	*CKM*
cg18094366	.01	.001	5.90e-06	22	24237343	*MIF*
cg24822696	.01	.002	6.48e-06	6	125283245	*RNF217*
cg26251101	.01	.003	6.64e-06	2	46768944	*RHOQ*
cg21888241	-.01	.001	1.39e-05	11	14541921	*PSMA1*
cg25829945	.03	.005	1.40e-05	3	159706383	*IL12A*
cg20969194	.04	.008	1.50e-05	2	223163175	*PAX3*
cg27106909	.04	.009	1.63e-05	16	30106897	*YPEL3*
cg18721212	-.02	.004	1.67e-05	19	36827430	*ZFP14*
cg03166236	-.05	.010	1.89e-05	2	7062351	*RNF144A*
cg15423613	.01	.001	2.07e-05	7	66093349	*KCTD7*
cg20026939	.04	.010	2.13e-05	18	44788898	NA
cg18026026	-.01	.002	2.35e-05	10	131265411	*MGMT*

Note: NA- not available

Table S4. Top CpG probes (p<1.00e-04) derived from the EWAS of prenatal maternal stress exposure in neonates, sorted by ascending p, in the ALSPAC sample (N = 828)

Probe name	*Effect*	*SE*	*p*	Chromosome	Position	Nearest gene
cg18383538	0,03	0,006	9,74E-06	6	28557823	NA
cg04166304	0,01	0,002	1,36E-05	14	45722617	*C14orf106*
cg03932361	0,06	0,014	1,88E-05	17	72667394	*RAB37*
cg00912926	0,08	0,018	2,42E-05	10	134775610	NA
cg13636189	0,02	0,006	5,26E-05	9	102587074	*NR4A3*
cg24446586	0,01	0,004	5,69E-05	7	27225078	*HOXA11AS*
cg26291519	-0,08	0,019	6,43E-05	15	76640005	NA
cg01978368	0,01	0,002	6,64E-05	1	16563189	*C1orf89*
cg23335255	-0,05	0,012	8,41E-05	19	37858855	NA
cg17319136	0,02	0,006	8,67E-05	6	32098233	*FKBPL*
cg22856512	0,01	0,003	8,91E-05	2	217497890	*IGFBP2*
cg08979136	0,01	0,003	8,95E-05	5	114504983	*TRIM36*
cg06362985	-0,06	0,016	9,41E-05	12	70132208	*RAB3IP*
cg20011562	0,01	0,003	9,98E-05	6	12749352	*PHACTR1*

Note: NA- not available

Table S5. Top CpG probes (meta p<1.00e-04) derived from the EWAS meta-analysis of prenatal maternal stress exposure in neonates, sorted by ascending meta p, in the fixed-effects EWAS meta-analysis (N = 1,740)

Probe name	Chromosome	Position	Effect (SE)	Direction [*]	Meta *P*	Heterogeneity *P*	Nearest gene (s)
cg13529437	6	43607635	-0.04 (.007)	--	1.00e-06	.47	*MAD2L1BP*
cg01978368	1	16563189	0.01 (.002)	++	3.48e-06	.78	*C1orf89*
cg04129946	2	201753996	0.02 (.004)	+	7.59e-06	.73	*PPIL3;NIF3L1*
cg20959676	1	23696021	0.01 (.003)	++	8.67e-06	.09	*C1orf213;ZNF436*
cg17631424	4	69312514	-0.06 (.013)	--	1.13e-05	.81	*TMPRSS11E*
cg19459675	4	166249239	0.02 (.004)	++	1.59e-05	.76	*SC4MOL*
cg12947485	4	25310668	-0.04 (.009)	--	1.71e-05	.75	NA
cg20011562	6	12749352	0.01 (.002)	++	1.85e-05	.18	*PHACTR1*
cg02644494	19	6412686	0.03 (.006)	++	1.88e-05	.82	*PVRL1*
cg01686933	11	119596104	-0.02 (.006)	--	2.12e-05	.81	NA
cg00409356	5	1879525	0.04 (.009)	++	2.17e-05	.17	*IRX4*
cg17332603	4	7326576	0.03 (.008)	++	2.19e-05	.35	*SORCS2*
cg15150970	2	25473529	0.03 (.007)	++	2.44e-05	.55	*DNMT3A*
cg08272572	19	35953646	0.01 (.003)	++	2.47e-05	.32	NA
cg01625242	18	56886915	0.04 (.009)	++	2.62e-05	.87	*GRP*
cg27518692	7	61627470	0.02 (.003)	++	2.67e-05	.49	*DCAF7*
cg27106909	16	30106897	0.03 (.008)	++	2.79e-05	.08	*YPEL3*
cg02011374	19	1761780	0.04 (.008)	++	2.81e-06	.79	*ONECUT3*
cg14783581	3	137482478	0.03 (.006)	++	2.81e-05	.24	*SOX14*
cg04921109	14	69952104	0.04 (.010)	++	2.86e-05	.25	*FLJ44817*
cg26548653	19	42829042	0.02 (.005)	++	3.08e-05	.91	*MEGF8;TMEM145*
cg00716660	1	224543513	-0.03 (.007)	--	3.15e-05	.31	*CNIH4*
cg19732144	5	166403816	-0.03 (.006)	--	3.21e-05	.68	NA
cg19227710	3	151285666	-0.04 (.010)	--	3.28e-05	.80	NA
cg09314421	8	21771252	0.01 (.003)	++	3.42e-05	.36	*DOK2*
cg13722419	2	160088036	-0.05 (.012)	--	3.46e-05	.72	*TANC1*
cg09972192	12	72667326	0.02 (.006)	++	3.72e-05	.29	*LOC283392;TRHDE*
cg14620593	17	10326392	-0.057 (.014)	--	3.92e-05	.80	*MYH8*
cg08339172	17	63692987	-0.05 (.012)	--	9.30e-05	.85	*CCDC46*
cg20759626	13	74250870	-0.03 (.008)	--	4.30e-05	.74	NA
cg11442280	2	164461604	-0.03 (.008)	--	4.72e-05	.35	NA
cg00321480	8	143533853	0.02 (.004)	++	4.75e-05	.93	NA
ch.X.16390	X	113225430	0.03 (.007)	++	4.96e-05	.11	NA
cg03224850	1	19199155	-0.05 (.013)	--	5.01e-05	.31	*ALDH4A1*
cg05012697	15	88798331	0.03 (.006)	++	5.50e-05	.68	*NTRK3*
cg22146312	19	47852819	0.02 (.005)	++	5.78e-05	.33	*DHX34*
cg13332172	19	37178884	0.02 (.004)	++	5.85e-05	.80	*ZNF567*
cg16670155	19	18557220	-0.01 (.002)	--	6.02e-05	.53	*ELL*
cg02760218	6	31866586	0.05 (.014)	++	6.10e-05	.13	*EHMT2*
cg24620673	4	66535655	0.03 (.007)	++	6.26e-05	.42	*EPHA5*
cg11382417	11	41481655	0.03 (.009)	++	7.03e-05	.88	NA
cg17087669	6	36099123	0.03 (.008)	++	7.11e-05	.38	*MAPK13*
cg15558675	3	195163052	0.02 (.004)	++	7.30e-05	.87	*ACAP2*
cg18260343	3	50606637	0.01 (.003)	++	7.31e-05	.85	*HEMK1;C3orf18*
cg18947995	14	101350739	-0.01 (.004)	--	7.34e-05	.06	*MIR136;RTL1*
cg23998381	2	73114610	0.02 (.004)	++	7.53e-05	.10	*SPR*
cg07515250	8	141873509	-0.03 (.007)	--	7.69e-05	.91	*PTK2*
cg23033906	14	21769332	0.01 (.002)	++	7.81e-05	.30	*RPGRIP1*
cg26821498	1	28415421	0.02 (.005)	++	7.84e-05	.95	*EYA3*
cg18588052	6	111408752	0.01 (.003)	++	8.26e-05	.58	*SLC16A10*
cg15867197	19	46196291	0.01 (.003)	++	8.28e-05	.82	*QPCTL;SNRPD2*
cg00682734	20	55200973	0.03 (.008)	++	8.43e-05	.80	NA
cg14817758	11	32914605	-0.01 (.002)	--	8.53e-05	.42	*QSER1*
cg03995156	6	32122864	0.03 (.008)	++	8.73e-05	.42	*PPT2*
cg18026026	10	131265411	-0.01 (.002)	--	9.46e-05	.36	*MGMT*
cg15233611	12	122244660	0.03 (.008)	++	9.50e-05	.08	*SETD1B*
cg12359592	3	138048777	0.01 (.001)	++	9.73e-05	.81	*TXNDC6*
cg23207305	11	15012901	-0.03 (.009)	--	9.77e-05	.86	NA
cg09579151	12	41043074	-0.02 (.005)	--	9.88e-05	.67	NA

[*] input order: ALSPAC, Generation R

Note: NA- not available

Table S6. Top CpG probes (meta p<1.00e-04) derived from the EWAS meta-analysis of prenatal maternal stress exposure in neonates, sorted by ascending meta p, in the random-effects EWAS meta-analysis (N = 1,740)

Probe name	Chromosome	Position	Effect(SE)	Direction*	Meta P	Heterogeneity P	Nearest gene(s)
cg13529437	6	43607635	-0.04 (.007)	--	1.00e-06	.47	*MAD2L1BP*
cg02011374	19	1761780	0.04 (.008)	++	2.81e-06	.79	*ONECUT3*
cg01978368	1	16563189	0.01 (.002)	++	3.48e-06	.78	*C1orf89*
cg04129946	2	201753996	0.02 (.004)	++	7.58e-06	.73	*PPIL3;NIF3L1*
cg18026026	10	131265411	-0.01 (.002)	--	9.46e-06	.36	*MGMT*
cg17631424	4	69312514	-0.06(.013)	--	1.13e-05	.81	*TMPRSS11E*
cg19459675	4	166249239	0.02 (.004)	++	1.59e-05	.77	*SC4MOL*
cg12947485	4	25310668	-0.04 (.009)	--	1.71e-05	.74	NA
cg02644494	19	6412686	0.03 (.006)	++	1.88e-05	.81	NA
cg01686933	11	119596104	-0.02 (.006)	--	2.12e-05	.80	*PVRL1*
cg17332603	4	7326576	0.03 (.008)	++	2.18e-05	.35	*SORCS2*
cg15150970	2	25473529	0.03 (.007)	++	2.44e-05	.55	*DNMT3A*
cg08272572	19	35953646	0.01 (.003)	++	2.47e-05	.32	*NA*
cg01625242	18	56886915	0.04 (.008)	++	2.62e-05	.87	*GRP*
cg27518692	17	61627470	0.01 (.003)	++	2.67e-05	.49	*DCAF7*
cg26548653	19	42829042	0.02 (.005)	++	3.08e-05	.91	*MEGF8;TMEM145*
cg19732144	5	166403816	-0.03 (.006)	--	3.21e-05	.68	*NA*
cg19227710	3	151285666	-0.04 (.010)	--	3.28e-05	.80	*NA*
cg09314421	8	21771252	0.01 (.003)	++	3.41e-05	.36	*DOK2*
cg13722419	2	160088036	-0.05 (0.01)	--	3.46e-05	.72	*TANC1*
cg14620593	17	10326392	-0.06 (.014)	--	3.92e-05	.80	*MYH8*
cg20759626	13	74250870	-0.03 (.008)	--	4.30e-05	.74	*NA*
cg11442280	2	164461604	-0.03 (.008)	--	4.70e-05	.35	*NA*
cg00321480	8	143533853	0.02 (.004)	++	4.74e-05	.93	*NA*
cg00716660	1	224543513	-0.03 (.007)	--	5.33e-05	.31	*CNIH4*
cg05012697	15	88798331	0.03 (.006)	++	5.50e-05	.68	*NTRK3*
cg22146312	19	47852819	0.02 (.005)	++	5.78e-05	.33	*DHX34*
cg13332172	19	37178884	0.02 (.004)	++	5.85e-05	.80	*ZNF567*
cg16670155	19	18557220	-0.01 (.002)	--	6.02e-05	.53	*ELL*
cg24620673	4	66535655	0.03 (.007)	++	6.26e-05	.42	*EPHA5*
cg11382417	11	41481655	0.03 (.009)	++	7.02e-05	.89	*NA*
cg17087669	6	36099123	0.03 (.008)	++	7.11e-05	.38	*MAPK13*
cg15558675	3	195163052	0.02 (.004)	++	7.30e-05	.87	*ACAP2*
cg18260343	3	50606637	0.01 (.003)	++	7.30e-05	.85	*HEMK1;C3orf18*
cg03224850	1	19199155	-0.05 (.013)	--	7.61e-05	.91	*ALDH4A1*
cg07515250	8	141873509	-0.03 (.007)	--	7.68e-05	.91	*PTK2*
cg26821498	1	28415421	0.02 (.005)	++	7.83e-05	.95	*EYA3*
cg18588052	6	111408752	0.01 (.002)	++	8.26e-05	.58	*SLC16A10*
cg15867197	19	46196291	0.01 (.003)	++	8.28e-05	.82	*SNRPD2;QPCTL*
cg00682734	20	55200973	0.03 (.008)	++	8.43e-05	.80	*NA*
cg14817758	11	32914605	-0.01 (.002)	--	8.53e-05	.42	*QSER1*
cg03995156	6	32122864	0.03 (.008)	++	8.73e-05	.42	*PPT2*
cg02907021	1	230986696	-0.02 (.005)	--	9.13e-05	.42	*C1orf198*
cg08339172	17	63692987	-0.05 (.012)	--	9.30e-05	.85	*CCDC46*
cg12359592	3	138048777	0.01 (.001)	++	9.73e-05	.81	*TXNDC6*
cg23207305	11	15012901	-0.04 (.009)	--	9.78e-05	.86	*NA*
cg09579151	12	41043074	-0.02 (.005)	--	9.89e-05	.68	*NA*

* input order: ALSPAC, GEN-R

Note: NA= not available

Table S7. Annotation of top DMRs associated with gPMSE score in cord blood, in the Generation R sample (N = 912)

Cluster Name	CpG probes	Chromosome	Position	Nearest gene(s)
90946	cg09141953	20	62948235	*MYT1;LINC00266-1;CICP4*
	cg19192585	20	62948037	*MYT1;LINC00266-1;CICP4*
	cg24189721	20	62948134	*MYT1;LINC00266-1;CICP4*
135488	cg07355590	7	133812072	*LRGUK*
	cg10839521	7	133811911	*LRGUK*
	cg12531972	7	133811940	*LRGUK*
	cg14455998	7	133811808	*LRGUK*
	cg14706178	7	133812217	*LRGUK*
	cg15280728	7	133812269	*LRGUK*
	cg15955731	7	133812369	*LRGUK*
	cg16776231	7	133812041	*LRGUK*
	cg17676607	7	133811837	*LRGUK*
	cg19967800	7	133811828	*LRGUK*
	cg22578125	7	133812031	*LRGUK*
66361	cg01906998	17	73974972	*ACOX1; C17orf106*
	cg02701084	17	73975226	*ACOX1;C17orf106*
	cg02760553	17	73975121	*ACOX1;C17orf106*
	cg10931252	17	73975914	*ACOX1; C17orf106*
	cg11507926	17	73975697	*ACOX1; C17orf106*
	cg12452675	17	73975215	*ACOX1*
	cg14770570	17	73975813	*ACOX1*
	cg15269548	17	73975372	*ACOX1*
	cg16419345	17	73976089	*ACOX1;C17orf106*
	cg19198791	17	73975107	*ACOX1;C17orf106*
	cg19652678	17	73975160	*ACOX1; C17orf106*
	cg21356179	17	73974861	*ACOX1; C17orf106*
	cg21697561	17	73974931	*ACOX1; C17orf106*
	cg24015057	17	73975129	*ACOX1; C17orf106*
	cg24357503	17	73975484	*ACOX1; C17orf106*
